# A Novel Wearable Flexible Dry Electrode Based on Cowhide for ECG Measurement

**DOI:** 10.3390/bios11040101

**Published:** 2021-04-01

**Authors:** Yiping Huang, Yatong Song, Li Gou, Yuanwen Zou

**Affiliations:** College of Biomedical Engineering, Sichuan University, Chengdu 610065, China; hyp@stu.scu.edu.cn (Y.H.); songyatong@stu.scu.edu.cn (Y.S.); gouli@scu.edu.cn (L.G.)

**Keywords:** wearable, flexible dry electrode, natural leather, plasma sputtering, skin-electrode impedance

## Abstract

The electrocardiogram (ECG) electrode, as a sensor, is an important part of the wearable ECG monitoring device. Natural leather is rarely used as the electrode substrate. In this paper, wearable flexible silver electrodes based on cowhide were prepared by sputtering and brush-painting. A signal generator, oscilloscope, impedance test instrument, and ECG monitor were used to build the test platform evaluating the performance of electrodes with six subjects. The lossless waveform transmission can be achieved with our electrodes. Therefore, the Pearson’s correlation coefficient calculated with input waveform and output waveform of the electrodes based on the top grain layer (GLE) and the split layer (SLE) of cowhide were 0.997 and 0.998 at 0.1 Hz respectively. The skin electrode impedance (Z) was tested, and the parameters of the equivalent circuit model of the skin electrode interface were calculated by a fitting method, indicating that the Z of the prepared electrodes was comparable with the standard gel electrode when the skin is moist enough. The signal-to-noise ratio of the ECG of the GLE and the SLE were 1.148 and 1.205 times that of the standard electrode in the standing posture, which meant the ECG measured by our electrodes was basically consistent with that measured by the standard electrode.

## 1. Introduction

Cardiovascular diseases (CVDs) are currently the leading cause of death globally [1]. Long-term effective monitoring of electrocardiogram (ECG) can detect most CVDs. Wearable devices for ECG monitoring can realize long-term effective monitoring of ECG [2,3,4]. The ECG signal quality measured with wearable devices within 24 h can be comparable to that of Holter systems, which are used for clinical ambulatory ECG recording [5,6,7,8]. However, more researches should be done to assess ECG signal quality measured with wearable devices over 24 h taking the long-term monitoring of wearable devices into consideration.

The ECG electrode is an important part of wearable ECG monitoring device, which can be divided into the standard Ag/AgCl electrode, micro-needle electrode, non-contact electrode and flexible dry electrode. Currently, the standard Ag/AgCl electrode with conductive gel is the most commonly used electrode for ECG measurement in clinic, which has some problems, such as limited shelf life, sensitization, and cumbersome skin treatment in use [9]. Microneedle electrodes need to pass through the stratum corneum of the skin, which can cause some degree of injury or discomfort to the human body. The polymeric micro-needle electrode made by Conor O’Mahony et al. belongs to this category [10]. He Zhang et al. prepared a flexible micro-needle electrode based on PDMS to record ECG in daily life. Although it did not cause human discomfort, it still needed to puncture the skin surface [11]. A non-contact electrode realizes the measurement of human physiological electrical signals through capacitance without contact [12], which is the current frontier of electrode research, however, the electrode is not commonly used due to the limitations of technology and materials. The flexible capacitive ECG measurement electrode based on MEMS prepared by Long-Fei Wang et al. [13] is such an electrode.

Flexible electrodes without micro-needles are the most commonly used in wearable devices for ECG monitoring. The flexible electrode allows as much contact area with the skin as possible through its flexibility, thus reducing impedance and yielding high-quality ECG signals. ECG measured by flexible electrodes are consistent with the standard Ag/AgCl electrodes. There is no risk of sensitization or skin treatment in the process of ECG measurement. Flexible electrodes can be fabricated utilizing a variety of different technologies, such as conventional printing technology [14,15], high-resolution lithography patterning [16], electrostatic flocking technology [17], and so on. At present, more and more flexible electrodes can measure multiple physiological signals simultaneously [18,19,20,21,22]. Fused sensor information helps the doctor make a more accurate diagnosis [23]. Among them the most representative one is the graphene electronic tattoo sensors fabricated by Shideh Kabiri Ameri et al. [24]. Flexible electrodes usually consist of flexible substrate and conductive layer, sometimes adhesive layer is also needed. The conductive adhesive layer in some flexible dry electrodes, fusing the conductive and adhesive layer, can offer stable skin-electrode contact to decrease motion artifacts [25,26]. Moreover, the plastic hydrogel-based electronic skin fabricated by Xiaofeng Pan et al. fused all the three layers and was completely soft and deformable [19].

There are also disadvantages in flexible electrodes. For example, the quality and reliability of recorded ECG signals by wearable devices are more sensitive to different factors such as electrode placement, skin humidity, user activities, and contact pressure [27]. Unfortunately, there has not been much research and uniform standard for these factors. Pricewise, although there are some low-cost wearable devices with excellent performance [28], most of the available wearable devices which can measure clinical-grade ECG fall within the price range of luxury products [29]. Also, widely accepted excellent wearable devices products are not readily available on the market and in clinic although there are many products that can be used in laboratory environments. Otherwise, the influence of washing processes, temperature, sweat, moisture, mechanical impacts, repeated bending and compression, and light (especially sunlight) should be carefully considered [30]. The ECG measurements during walking or other intense sports are always affected by the motion artifacts [31].

There are many kinds of substrate materials for flexible dry electrodes, such as carbon-based materials, artificial fabric [32], papers [33] and so on. However, natural leather material is rarely used as the substrate. Natural materials have advantages such as simple processing, high availability and cheap price etc. As a natural material, cowhide not only has excellent properties such as high tensile strength, tear strength and denaturation temperature [34]. Moreover, as a common fashion element of clothing [35], it is easily integrated into wearable devices. In addition, cowhide cleaning is simple, and scrubbing is the main way to avoid possible damage to the electrodes caused by cleaning. Therefore, the natural material cowhide is used as the substrate of flexible dry electrode. Like other wearable devices, there is still no universally recognized evaluation standard for flexible dry electrodes, which is a serious problem for products that contact human skin for a long time [36]. Obviously, the standard disposable Ag/AgCl electrode evaluation standard is not fully applicable to wearable device sensors for long-term ECG monitoring.

In this paper, novel flexible electrodes were prepared, and the natural leather material cowhide was used as the flexible substrate to demonstrate the potential of the cowhide for wearable electronic applications. Three experiments were designed to evaluate the prepared electrodes so that we can find the differences in performances between the flexible electrodes based on cowhide and the standard Ag/AgCl electrode used in clinic.

## 2. Materials and Methods

### 2.1. The Fabrication of Electrodes

**Figure 1:**
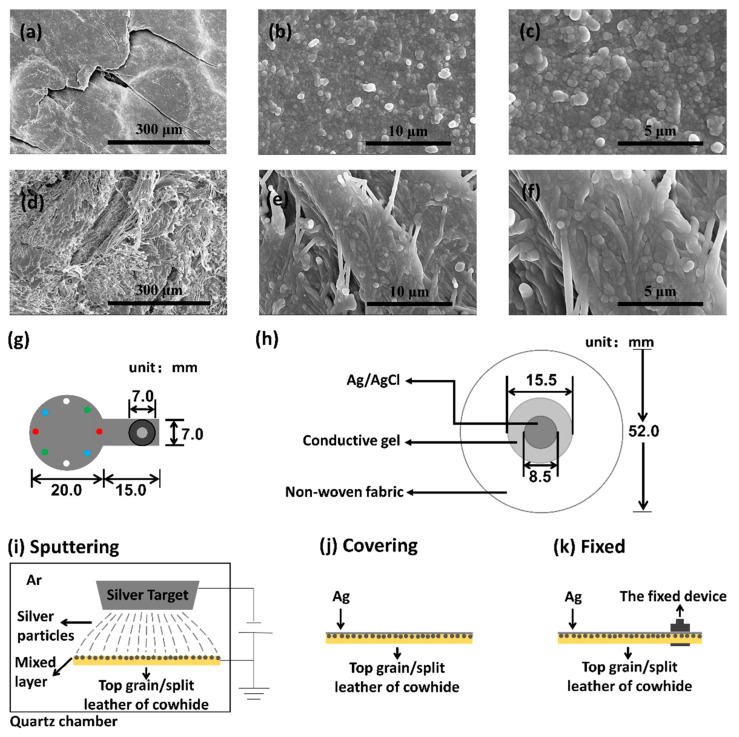
(**a**–**f**) SEM images of surface of cowhide ((**a**–**c**) the top grain layer and (**d**–**f**) the split layer). (**g**) Schematic diagrams of the prepared electrodes, the color points are used to indicate the position of impedance measurement when characterizing the uniformity of silver paste. The same color points are a pair, but there are no such points on the actual electrode. (**h**) Schematic diagrams of the standard Ag/AgCl electrode. (**i**–**k**) Schematic diagram of sputtering, covering silver paste and installation of fixed device in fabrication of electrodes.

Our prepared electrodes had two layers, the flexible substrate and the conductive layer. As mentioned in introduction, cowhide not only has excellent flexibility, but also has the advantages of mature processing technology, excellent mechanical properties, high availability, low price and so on. Moreover, leather is used in garment making for long time. Leather garments are often regarded as a symbol of fashion and personality. Compared with polymer material substrate, leather is easier to be made into wearable products like smart clothing. The cleaning method of leather products is mild, which can greatly avoid the damage to the conductivity of electrodes caused by cleaning compared with artificial fabric. So, the flexible substrate material in this experiment was cowhide commercially purchased from an online store in Jiaxing, Zhejiang, and protein was the essential composition of it. Intuitively, the split layer of cowhide is fluffier and rougher than the top grain layer. Figure 1a–c,d–f are the scanning electron microscope (SEM) images of the surfaces of the top grain layer and the split layer respectively. As depicted in Figure 1a–c, the surface pattern of the top grain layer is much more regular than that of the split layer of cowhide. So, we decided to use the top grain layer and the split layer as flexible substrates to make electrodes respectively. Silver, which has excellent conductivity and is often used for physiological signal detection, was selected as the conductive layer material.

The shape of the prepared electrodes is shown in Figure 1g, in which the circular part with a diameter of 20.0 mm and the rectangular part on the right was 15.0 mm long and 7.0 mm wide. The electrical signals are detected by the circular part which was designed according to the study of Flurin Stauffer et al. [26]. The detected electrical signal is transmitted to the signal processing circuit through the rectangular part to avoid possible interference caused by welding conducting wires on the electrode surface.

A conductive thin film was formed on the surface of cowhide by plasma sputtering. Figure 1h shows the structural diagram of the standard Ag/AgCl electrode. The Ag/AgCl circular sheet of diameter 8.5 mm is fixed on the non-woven fabric with diameter of 52.0 mm, and the conductive gel with diameter 15.5 mm is covered on the sheet. The conductive gel directly contacted with human skin. The silver nanoparticles were sputtered on the surfaces of substrates with plasma sputtering instrument VTC-16-3HD from Kejing Materials Technology Co., LTD, Anhui, China. Figure 1i shows the sputtering process, which lasted only for 10 min to avoid the damage to cowhide caused by the high temperature due to long-term work. Considering that the sputtering time is not long, in order to ensure that the conductivity of the electrode, the surface was then brush-painted with silver paste bought from Sunrise Electronic Materials Co., Ltd., Shanghai, China, as shown in Figure 1j. Furthermore, conductivity of the electrode was measured to observe uniformity of the silver paste. If the silver paste is not brushed uniformly, the resistance difference in different directions will exceed the normal range. Considering the size of the electrodes, four groups of points were selected to measure the impedance every 45 degrees to observe the difference of impedance in different directions. As shown in Figure 1g, the resistances were measured at the red, blue, white, and green points (the main purpose of these points is to show the position of measurement, there are no such points on the prepared electrodes) with digital multimeter UT890D from UNI-T in China. Each group was measured 3 times and the average value was calculated and presented. The average value (AVG) and the standard deviation (SD) of the four measured resistance values were calculated to evaluate the resistance difference in different directions. The smaller the SD was, the smaller the difference was, the more uniform the sliver paste was. Finally, in order to connect the ECG monitoring device, the rear end of the conductive part of the electrode was installed with a fastener snap, as shown in Figure 1g,k.

### 2.2. Signal Transmission Experiment

**Figure 2:**
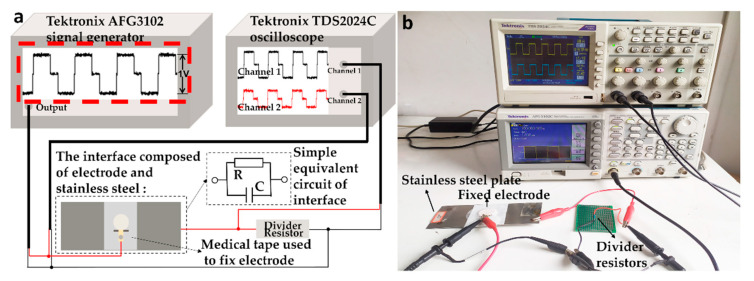
(**a**) The schematic diagram, the test circuit is composed of signal generator, electrode-stainless steel interface, divider resistor and oscilloscope. (The picture on the oscilloscope is only the demo of a certain result, and all the detailed results are shown in the result section.) and (**b**) The experimental scene of the signal transmission experiment.

As the sensor of ECG monitoring equipment, the key function of electrode is to transmit physiological electrical signals losslessly. The lossless property in signal transmission of the electrodes was evaluated by signal transmission experiment. The lossless property in signal transmission in this paper refers to the shape of the electric signal is not distorted in the time domain and the passband of signal does not change in the frequency domain after passing through the interface composed of electrode and other substances. Biphasic pulse can reduce charge accumulation (avoid electrode polarization phenomenon) and contain abundant harmonic components, which is conducive to the detection of signal loss in the frequency domain. Therefore, we choose a fixed biphasic pulse signal as the input signal. The peak-to-peak value of the signal is 1 V, and the shape is shown in the red dotted box in Figure 2a. We chose stainless steel as the other side of the interface. This is because the interface formed by the stainless steel is stable, unnecessary capacitive reactance and inductive reactance interference will not be introduced, and the stainless steel has good conductivity, which can highlight the transfer performance caused by the electrode itself rather than the stainless steel.

Figure 2a is the schematic diagram. Figure 2b shows the experimental scene diagram. The experiment circuit included a signal generator, the interface of electrode and stainless steel, a voltage divider and an oscilloscope. The former three were connected in series to form the circuit, and the oscilloscope was connected in parallel to the divider. Tektronix AFG3102 signal generator generated the biphasic pulse signals with frequencies of 0.01 Hz, 0.1 Hz, 1 Hz, 10 Hz, 100 Hz, and 1000 Hz respectively because physiological electrical signals are basically distributed in the frequency range. The tested electrode was fixed on the center of a 140 mm × 60 mm stainless steel plate with the medical tape to form the interface of electrode and stainless steel, which is connected with a voltage divider in series in the circuit. As seen in Figure 2a,b, the rectangular part of the electrode, which transmitted the signal, protruded from the gap of the medical tape cut in advance, which can not only effectively fix the electrode, but also facilitate the connection of subsequent equipment. The simple equivalent circuit of the interface is shown in Figure 2b. The value of the voltage divider was adjustable to obtain the output waveform with appropriate peak-peak value (at least 400 mV). A Tektronix TDS2024C oscilloscope connected in parallel on the divider resistor displayed the output waveform. In order to quantitatively analyze the changes of input and output signals after passing through the interface of electrode and stainless steel, the Pearson’s correlation coefficient (PCC) of input S_i_ and output signal S_o_ were calculated with MATLB 2018B. The calculation formula is as follows:

PCC = (∑ (S_i_ − S_i_) (S_o_ − S_o_))/√ (∑ (S_i_ − S_i_)^2^ ∑ (S_o_ − S_o_)^2^) (1)

where S_i_ and S_o_ represent the average value of S_i_ and S_o_ respectively. The closer the value of PCC is to 1, the smaller the difference between input signal and output signal is, and the better the lossless property of signal transmission of electrode is.

Apart from the Pearson’s correlation coefficient, the least-mean-squared error (LMSE), which shows the differences between the original and output waveforms in the time domain in absolute units (V) was calculated. In this experiment, the changes of signal shape and passband were underlined, rather than the change of amplitude that can be effectively solved by subsequent amplification circuit. Therefore, the linear conversion method in the normalization is used to convert the output waveform to the range of −0.5 V to 0.5 V (the voltage range of input signal). Then, the LMSE was calculated according to the following formula.

LMSE = (∑ (S_i_ − S_o_’)^2^)/n (2)

The S_o_’ represents the value of S_o_ after conversion. The n represents the number of the values of S_o_. Opposite to PCC, the closer the LMSE is to 0, the better the lossless property of signal transmission of electrode is.

In order to further clarify the difference of original and output waveform in frequency, the relative change value R of Fast Fourier Transform (FFT) frequency is calculated by original signal S_i_ and the output signal S_o_ using MATLB 2019B. The calculation process is as follows:

R = log ((FFT(S_i_) − FFT(S_o_))/(FFT(S_i_))). (3)

The FFT transformation points are 2048. The difference value between input signal and output signal after FFT is the most intuitive manifestation of the difference between input signal and output signal in frequency domain. In order to further eliminate the influence caused by the size of the value of the signal itself, the relative difference obtained by dividing by the value of the input signal can effectively measure the difference of the input and output signals in the frequency domain. When the value of R is close to 0, the signal change in the frequency domain is small, and the signal transmission performance of the electrode is excellent.

### 2.3. Skin-Electrode Impedance Test

The skin-electrode impedance test reflected the performance of the electrode by measuring the impedance of the skin-electrode interface. The stratum corneum (SC) is a sturdy protective barrier made up of dead cells that keep most bacteria and viruses out of the human body. The resistance of stratum corneum is much larger than that of other tissues in the body. Therefore, the impedance of skin-electrode interface is the main impedance in ECG measurement, and its value is of great significance [37,38]. If the value is too large, the measured signal amplitude may decrease, and some signal details are lost. So, the low skin-electrode impedance was expected. Theoretically, we hope that the skin electrode impedance will not change with the change of frequency, so that the measurement results will be more stable and reliable. In other words, we hope that the skin electrode impedance is purely resistive. So, a low phase was what we expected. The impedance–frequency diagram and phase–frequency diagram were given in Section 3.3. Skin-Electrode Impedance Test.

**Figure 3:**
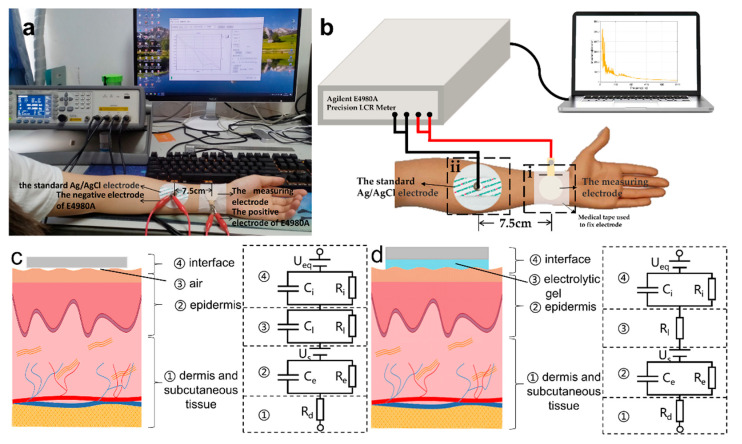
(**a**) A photo in measuring skin-electrode impedance. (**b**) The schematic of the experiment (the curve on the laptop screen is only a schematic diagram). Equivalent circuit diagram of (**c**) Dry electrode and (**d**) Wet electrode.

In this experiment, the skin-electrode impedance was measured with the E4980A precision LCR meter from Agilent. There are six subjects (three male and three female) in the test. The skin was not pretreated before the experiment. Two electrodes were placed on the left arm. Figure 3a demonstrates the scene of impedance test and Figure 3b shows the schematic of the experiment. We made the connection according to the manual of the E4980A precision LCR meter. As shown in Figure 3b(ii), the standard Ag/AgCl electrode was used as the auxiliary electrode to help form the circuit. The electrode in Figure 3b(i) was the measuring electrode fixed by the medical tape with the way mentioned in Section 2.2. Signal Transmission Experiment. In this experiment, the measuring electrode was one of the three electrodes: the two prepared electrodes based on cowhide and the standard Ag/AgCl electrode. The equivalent circuits of Figure 3b(i,ii) are shown in Figure 3c,d. The distance between the two electrodes was set to 7.5 cm. The common frequency range of physiological electrical signals is from 0.01 Hz to 1000 Hz, but 20 Hz is the lowest frequency the E4980A precision LCR meter can reach. So, the frequency changes from 20 Hz to 1000 Hz, with a sampling interval of 1 Hz. The measuring voltage was set to 1 V.

Furthermore, in order to explore a simple and effective method to reduce the skin electrode impedance, after the skin electrode impedance of the three electrodes are all finished, we tried to use common lotion bought from a local store (mainly composed of water and glycerin). We applied an appropriate amount of lotion on the arm area shown in Figure 3b(i), and then spread it evenly. A period of 10 min rest was adopted for the skin to fully absorb the lotion. Then, the electrode was fixed based on the top grain layer of cowhide and measured the skin electrode impedance again. After the measurement, we cleaned the area on the arm where the lotion was applied, gently wiped off the water, waited for a period of time for the skin to dry, and then measured the skin electrode impedance of the electrode based on the split layer in the above steps.

The equivalent circuit of skin-electrode interface can quantitatively describe the parameters of skin-electrode interface. Figure 3c,d are the equivalent circuit diagrams of dry electrode and wet electrode, respectively [39,40,41]. The equivalent circuit can be divided into four sections: ① dermal and subcutaneous tissue, ② epidermal layer, ③ air gap in Figure 3c and electrolyte in Figure 3d, and ④ skin-electrode interface from bottom to top. According to the measured data of impedance test and the equivalent circuit, the parameters on the right side of Figure 3c,d can be calculated with fitting method.

Z_1_, Z_2_, Z_3_, Z_4_ stand for the impedance of the ①, ②, ③, ④ section, respectively. Due to the two electrodes are close on subject’s arm, it can be assumed that the physiological potential of these areas is the same. Therefore, U_s_, U_eq_ are not calculated into the formula. According to the actual circuit connection in Figure 3b and equivalent circuit diagram in Figure 3c,d, the following expression can be obtained:

Z_1_ = R_d_, (4)

1/Z_2_ = 1/(1/j ω C_e_) + 1/R_e_, (5)

So, the expression of Z_2_ is as follows:

Z_2_ = R_e/_(1 + (2 π f R_e_ C_e_)^2^) − j (2 π f R_e_^2^ C_e_/(1 + (2 π f R_e_ C_e_)^2^)), (6)

Z_3_, Z_4_ can be obtained with the same method. The total impedance Z is expressed as follows:

Z = Z_1_ + Z_2_ + Z_3_ + Z_4_, (7)

The expression containing Z and f can be obtained by the expression (4)–(7). The various parameters of the equivalent circuit diagram can be solved with the expression and the measured impedance and frequency by fitting method using Curve Fitting Tool in MATLAB R2018B. Z_1_, Z_2_, Z_3_, Z_4_ are calculated by using the obtained parameter values, the expression (4)–(7) and taking f = 20 Hz. The goodness of fit is used to evaluate the fitting effect. The closer the goodness of fit is to 1, the better the fitting effect is. The formula of goodness R^2^ of fit is as follows.

R^2^ = (∑ (Z_i_’ − Z)^2^)/(∑ (Z_i_ − Z)^2^) (i = −1, 2, 3, …, n) (8)

where Z_i_ is the impedance values measured in the skin-electrode impedance test, n is the number of these values, $\bar{Z}$ is the average of the impedance values measured, and Z_i_’ is the fitting impedance values calculated by the various parameters solved with the expression (4)–(7).

### 2.4. ECG Test

High quality ECG measurement is extremely important for wearable ECG electrodes [42]. The quality of measured ECG signals can directly reflect the performance of electrodes in actual ECG measurement, which is an important standard to evaluate the performance of electrodes.

In this paper, ECG was measured with Epm10 physiological signal monitor of Mindray Company in China. There are six subjects (three male and three female) in the test. The purpose of this experiment is to prove that the electrodes based on cowhide have the potential to measure ECG with high quality comparable to that of the standard Ag/AgCl electrode. Our electrodes carried out ECG test firstly, and then disposable cotton swab was used to dip in appropriate amount of medical alcohol to wipe the area where the electrodes was placed. Lastly, the disposable ECG electrodes was used for ECG. The electrodes were placed on the subject’s left arm, right arm and left leg, respectively, and the electrode on the subject’s left arm was the positive electrode. In order to avoid introducing other variables in the measurement process, the positive electrode was the only one replaced each time. The prepared electrodes were fixed by an adjustable elastic bandage. In each experiment, the stretch length of the elastic bandage was the same to provide equal contact force between electrode and skin. Three typical daily postures—sitting, standing, and walking—were selected.

The measured original ECG data was processed with MATLAB R2018B as follows: first, the baseline drift was filtered by wavelet transform, then the EMG was filtered by low-pass filter with cut-off frequency of 60 Hz, and finally the 50 Hz power frequency interference was filtered by IIR trap with the cut-off frequencies of passband are 48.5 Hz and 51.5 Hz respectively [43,44,45]. The processed ECG measured with the standard Ag/AgCl electrodes in sitting posture were taken as reference ECG signal ECG_ref_ to calculate the signal-to-noise ratio (SNR) of each ECG, which is defined as the ratio of signal to noise. Reference ECG signal refers to the ECG signal that we regard as the ECG signal without noise. The higher the SNR is, the better the signal quality is. The calculation formula is as follows.

SNR = 10 × log ((∑ECG^2^)/(∑ (ECG − ECG_ref_)^2^)), (9)

Observation only in the time domain is certainly limited, so the ECG power spectrum, which is defined as the signal power per unit frequency band, is also used in the process of diagnosing the disease by ECG [46]. In this experiment, the power spectrum of the measured ECG was calculated with Blackman Window using MATLAB R2018B. P1 is the first peak except the near direct-current (DC) peak of the measured ECG power spectrum. On the basis of the theory of power spectrum of ECG, the frequency value of P1 multiplied by 60 equals the heart rate. Heart rate is one of the important characteristics of ECG [6].

## 3. Results

### 3.1. The Fabrication of Electrodes

**Figure 4:**
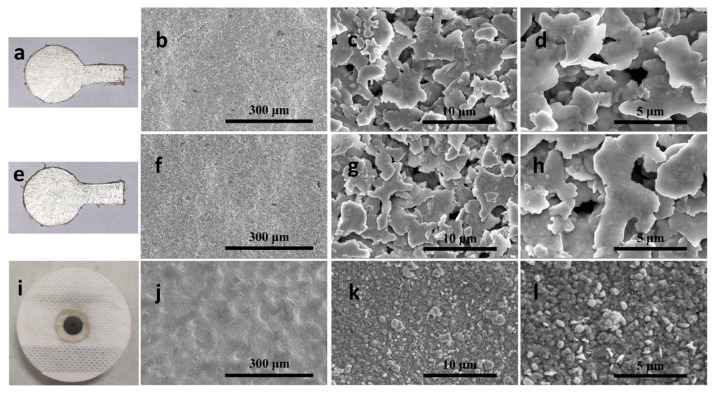
Photos and SEM images of electrodes (**a**–**d**) Electrode based on the top grain layer, (**e**–**h**) Electrode based on the split layer, (**i**–**l**) The standard Ag/AgCl electrode.

Figure 4a,e,i shows images of the prepared electrodes based on the top grain layer and the split layer without the fastener snap and the standard Ag/AgCl electrode. Compared with the standard Ag/AgCl electrode, these two electrodes can be sewn into clothing and there is no gel, which is more convenient and comfortable to wear. Unlike the standard Ag/AgCl electrode, the physiological signal detection part is separated from the signal transmission part, which can reduce the impact of the transmission part on the contact between the detection part and the human body. Figure 4b–d demonstrates the scanning electron microscope (SEM) images of the electrodes based on the top grain layer. Figure 4f–h are the scanning electron microscope (SEM) images belonging to the electrodes based on the split layer. Figure 4j–l are the scanning electron microscope (SEM) images belonging to the standard Ag/AgCl electrode. According to Figure 4, there was almost no difference between the surfaces of the electrodes based on the top grain and split layer although the two surfaces of cowhide are very different. The possible explanation is that we have adopted the same processing scheme for the two surfaces of cowhide. So, we need further experiments to explore other performances of the two electrodes. However, compared with the standard electrode, the surfaces of the electrode based on cowhide are smoother, but also looser. We take the standard Ag/AgCl electrode as the gold standard to judge whether the performances of the two prepared electrodes can meet the requirements of ECG measurement.

The experimental results of the resistance measurement for the assessment of conductivity and silver paste uniformity are shown in Table 1.

The experimental results of the resistance measurement for the assessment of silver paste uniformity are shown in Table 1. According to Table 1, the AVG of the resistance values of the four group points of the two electrodes based on cowhide were small compared with the resistance of some conductor, which indicates the conductivities of the two electrodes are good. The SD was not high compared with the measurement error, which means that the dispersion of resistance values in four directions was not high, and the uniformity of silver paste is good.

### 3.2. Signal Transmission Experiment

**Figure 5:**
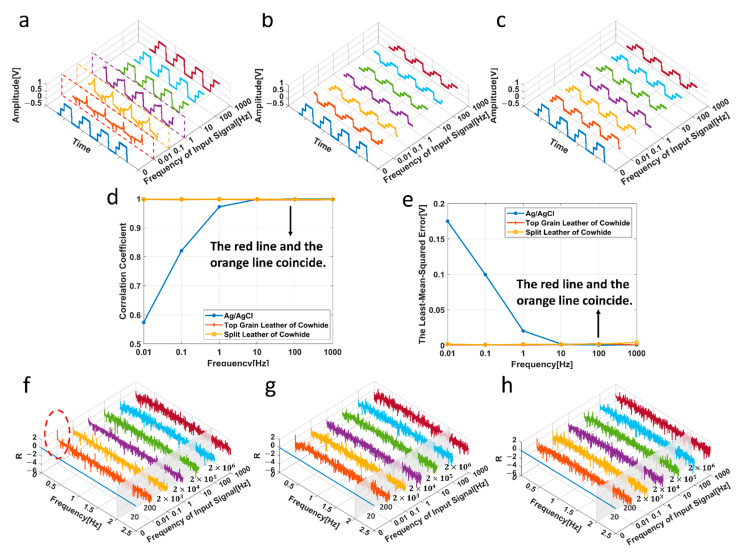
Experimental results of signal transmission:(**a**) The standard Ag/AgCl electrode (**b**,**c**) The electrodes based on the top grain layer and the split layer. (**d**) Pearson’s correlation coefficient of input and output waveform. (**e**) The least-mean-squared error of input and output waveform. FFT frequency relative variation diagram: (**f**) The standard Ag/AgCl electrode (**g**,**h**) The electrodes based on the top grain layer and the split layer.

Figure 5a–c shows the output waveform when the frequencies of the input biphasic pulse signals are 0.01 Hz, 0.1 Hz, 1 Hz, 10 Hz, 100 Hz and 1000 Hz, respectively (the signal graph at the frequency of 0Hz represents the original waveform). The *X*-axis represents the frequency of input signal. The *Y*-axis and the *Z*-axis are the time axis and the amplitude axis of the output waveform. Since frequency of the input signal is different, the time axis of the output signal is also inconsistent, so the specific time is not marked on the time axis. Figure 5d shows the result of PCC of input and output signals. The result of LMSE of input and output signals was shown in Figure 5e. A detailed analysis is discussed in Section 4.1.

The FFT frequency relative change (R) diagrams are obtained as shown in Figure 5f–h. The *X*-axis of Figure 5f–h is the frequency of the input signal, and the *Y*-axis and *Z*-axis represent the frequency in frequency domain and relative amplitude difference of the input signal and the output signal after FFT transformation, respectively.

### 3.3. Skin-Electrode Impedance Test

**Figure 6:**
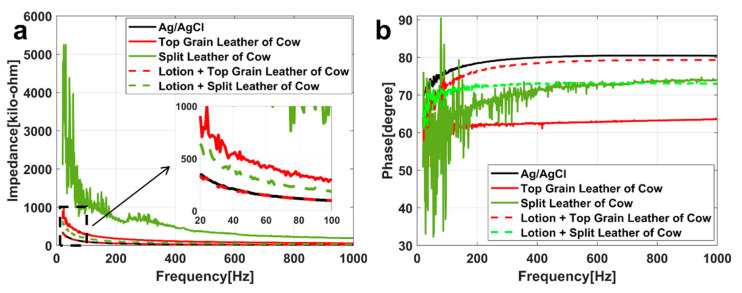
Diagram of (**a**) skin-electrode impedance-frequency and (**b**) skin-electrode phase-frequency of the first subject. The frequency range is 20–1000 Hz, each electrode is indicated by one color, and the application of lotion is indicated by a dotted line.

To minimize skin-related variations (thickness of SC, male–female physiological differences) among different subjects, the result of the skin-electrode measurements tested on one subject is shown and discussed. Therefore, subject-to-subject variations of skin-electrode impedance are not addressed [14]. The results of other subjects are shown in Supplementary Figures S1–S5. Figure 6a,b show the relationship between frequency and impedance, frequency and phase under different conditions (different electrodes and whether lotion is applied), respectively.

The black, red, and green lines in Figure 6 represent the data of standard Ag/AgCl electrode, the electrode based on the top grain layer and the electrode based on the split layer respectively. The dotted line represents the data of applying lotion. In Figure 6b, because the data of the electrode based on the split layer will be mixed together, green with different brightness is used to distinguish. A detailed discussion is provided in Section 4.2. The fitting results are shown in Table 2.

### 3.4. ECG Test

**Figure 7:**
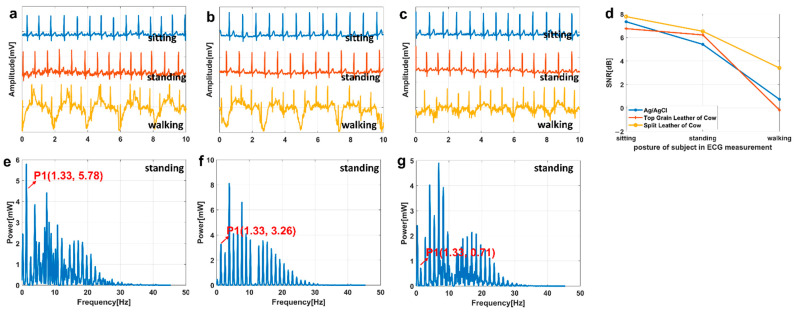
The ECG of (**a**) the standard Ag/AgCl electrode and (**b**,**c**) the electrodes based on the top grain layer and split layer. (**d**) Signal-to-noise ratio (SNR) diagram. The power spectrum of the standing posture of (**e**) the standard Ag/AgCl electrode (**f**) the electrode based on the top grain layer and (**g**) the electrode based on the split layer.

The result of the first subject is shown in Figure 7, and the results of other subjects are shown in Supplementary Figures S6–S10. Figure 7a–c are the measurement results of the standard Ag/AgCl electrode, the electrodes based on the top grain layer and the split layer, respectively. The measurement results of sitting posture, standing posture and walking posture are shown from top to bottom in each figure. Figure 7d shows the results of the signal-to-noise ratio (SNR) of each ECG.

Since the length of the paper, we will not show all the power spectrum here, and the power spectrum of standing posture of the three electrodes is selected for display and explanation. Figure 7e–g is the power spectrum of standing posture of the standard Ag/AgCl electrodes, the electrode based on the top grain layer and the split layer, respectively.

## 4. Discussion

At present, there are few studies on using natural leather as the material of ECG electrode. We proposed to use cowhide as the substrate of the ECG electrode, and designed experiments to prove its potential as the substrate material of ECG electrode, so as to provide another usable material for the development of wearable clothing apart from textiles.

However, some limitations should be noted. First, there were only six subjects (three males and three females) in the skin-electrode impedance and ECG test. Second, the subjects were all young people aged from 21 to 25. Third, limited by the accuracy of the test instrument, the frequency range of skin electrode impedance test is 20–1000 Hz. Fourth, the relationship between the surface roughness of the substrates and noise of the skin-electrode interface needs further exploration. At the same time, the relationship between the quality of ECG and the flexibility of the electrode, the force taken for fixation and the surface roughness of the electrodes also needs further exploration. Fifth, integrating electrodes into a garment will enable the long-term monitoring of bio-signals. However, continuous, effective, and real-time ECG monitoring needs equipment with high performance, and sufficient time is needed for subject recruitment, long-time ECG measurement and data processing. Therefore, we will complete the experiment in the next step. Sixth, concerning toxicity, cowhide and silver are common in daily life and do not show toxicity, but this study did not perform a biocompatibility test for human skin uses.

The following are discussions of the experimental results.

### 4.1. Signal Transmission Experiment

As can be seen from Figure 5d, the correlation coefficients of the standard Ag/AgCl electrode are far below 1 under 10 Hz while that of the electrodes based on cowhide are close to 1. In Figure 5a, the output waveform of the standard Ag/AgCl electrode (in dotted boxes) distorted until the frequency of input signal reaches 10 Hz. However, no serious distortion occurs in the waveform of the prepared electrodes in Figure 5b,c. The results of LMSE further proved this phenomenon in absolute units (V).

FFT transform difference graph (Figure 5f–h) can directly reflect the influence of electrode on different frequency components of waveform. Figure 5f–h show that the relative amplitude difference all fluctuates around 0. Only the standard Ag/AgCl electrode shows a high peak (in the red dotted circle) at the low frequency when the frequency of input signal is 0.01 Hz in Figure 5f, indicating that even though the standard Ag/AgCl electrode has a large distortion in the time domain, in fact, not much information is lost in the frequency domain. The high peak value is probably related to the conductive gel. After removing the conductive gel, the experimental results are consistent with those in Figure 5g,h.

It can be concluded that the signals passing through the standard Ag/AgCl electrode could have a certain degree of distortion in the time domain at low frequency (0.01–10 Hz), but there is only an obvious peak at 0.01 Hz in the frequency domain. The signals passing through the electrodes based on cowhide have no distortion in both the time domain and the frequency domain. Therefore, all the three electrodes can be considered to accurately transmit signals, and the electrodes based on cowhide have a slight advantage.

### 4.2. Skin-Electrode Impedance Test

In Figure 6a, both of the skin-electrode impedance of the prepared electrodes is higher than the standard Ag/AgCl electrode, and the impedance measured with the electrode based on the split layer can even reach about 10 times of that of the standard Ag/AgCl electrode. After applying the lotion, the impedance of the prepared electrodes was greatly reduced. In Figure 6b, the phase angle of the standard Ag/AgCl electrode was higher than that of the other two electrodes. Lotion produced a great influence on the phase angle of the electrode based on the top grain layer. After the application, the curve is close to the standard Ag/AgCl electrode. However, the influence on the phase angle of the electrode based on the split layer is smaller.

Since the surfaces of the prepared electrodes and the skin are not completely smooth, when they are in direct contact, there are many high-impedance air gaps, producing smaller capacitors between them. Therefore, when there is no conductive gel as the paste film between them, it is reasonable that the skin-electrode impedance is higher. After applying lotion, the moisture content of the skin is increased, the air gaps between the skin and electrode shrink or disappear, and the capacitance value decreases or becomes a lower resistance, so the total impedance decreases, and the phase angle increases. According to the experimental results, although there is a certain influence of the electrode material on the skin-electrode impedance, it is the contact interface type (dry electrode contact or wet electrode contact) of the skin-electrode produce a greater influence [47].

The goodness of fit in Table 2 are all above 0.90, indicating that the fitting effect is good, and these fitting parameters are a possibility of the actual situation. The difference between the five values of Z_1_ and Z_2_ is smaller due to the impedance of the dermis, subcutaneous tissue and epidermal layer which represented by Z_1_ and Z_2_ is almost constant in a short time. Z_3_ represents the impedance of air gap in the dry electrode equivalent circuit, and the impedance of conductive gel in the wet electrode. Obviously, the former is larger than the latter. Moreover, due to the difference in water absorption performance and tightness with skin between electrodes based on the top grain layer and the split layer, the size and number of air gaps formed in the skin-electrode interface are different. So, the difference between the five values of Z_3_ is larger. The difference between the five values of Z_4_ is larger because the conductive gel can effectively reduce the skin-electrode impedance. The lotion is equivalent to gel in the dry electrode, changing the mode of electrical conduction in skin-electrode interface. The value of Z is mainly determined by Z_4_, which proves that the decisive factor of skin-electrode impedance is the contact type of skin and electrode again.

In summary, although the skin-electrode impedance of the electrodes based on the top grain layer and the split layer are greater than that of the standard Ag/AgCl electrode, the problem can be successfully solved by simply applying lotion. The results of other subjects in Supplementary Figures S1–S5 have basically the same trend. Table 2 shows that the skin-electrode impedance of dry electrode is much greater than that of the wet electrode, likely because of the air gaps in the skin-electrode interface. Methods to solve the problem of air gaps, e.g., the high moisture level of the skin or the good fluidity of electrode material, could reduce the skin–electrode impedance effectively.

### 4.3. ECG Test

It can be seen from Figure 7d that in the sitting posture, SNR of the standard Ag/AgCl electrode and the electrode based on the split layer is basically the same, which is slightly higher than that of the electrode based on the top grain layer. The results difference of the three electrodes is likely to be caused by subtle changes of the subject’s state.

In the standing posture, in Figure 7d, the SNR of the electrode based on cowhide is basically the same, and that of the standard Ag/AgCl electrode is the lowest. In Figure 7a, the noise of the standard Ag/AgCl electrode is obviously stronger than those of the other two electrodes, because the electrodes based on cowhide are good flexible, and their shapes are changed with the shape of the skin.

In the walking posture, the SNR of the electrode based on the split layer is the highest, and the SNR of the electrode based on the top grain is the lowest in Figure 7d. The skin-electrode interface of the electrode based on the split layer may be more stable during the measurement process. So, the relative movement between the skin and the electrode was not as great as the other two. This may be related to the flexibility of the electrode, the forces and the contact area between the skin and the electrode, the roughness of the electrode surface, and so on.

In power spectrum of ECG, the larger the number of spectral lines in power spectrum is, the more the content (including noise and detailed information) is. In Figure 7e–g, the ECG signal measured by the standard Ag/AgCl electrode contains most content, and the electrodes based on the split layer contains the least content. According to Figure 7e–g, the heart rates of the subject are all 79.8 beats per minute, which is normal. Based on the Fourier theory, the P1 amplitude at some frequency in the power spectrum is related to the peak value of the original signal at that frequency. Then, the frequency of the highest peak of power spectrum should be larger than P1, the standard Ag/AgCl electrode has a lower value.

Above all, that the SNR of the ECG signals measured by the electrode based on the top grain layer and the split layer are consistent with that of the standard Ag/AgCl electrode. In the power spectrum, the ECG measured by all the three electrodes can reflect the signal characteristics. The results of other subjects in Supplementary Figures S6–S10 have the same characteristics. However, it is not a common feature that the noise of the ECG signals measured by our electrodes is lower than that of the standard Ag/AgCl electrode. The reasons for noise are complex, and changes may be caused by many factors.

## 5. Conclusions

In this paper, two flexible electrodes based on the top grain layer and the split layer of cowhide were designed and prepared to fabricate as wearable ECG monitoring sensors. The performance of these two electrodes and the standard Ag/AgCl electrode was evaluated by signal transmission experiment, skin-electrode impedance test and electrocardiogram (ECG) test. In the signal transmission experiment, the correlation coefficients of the standard Ag/AgCl electrode are well under 10 Hz (it means severe distortion) while that of the electrodes based on cowhide are close to 1 in the time domain. However, in the frequency, only the standard Ag/AgCl electrode shows a high peak at the low frequency when the frequency of input signal is 0.01 Hz. So, all the three electrodes can accurately transmit signals. However, the signals of the standard Ag/AgCl electrode had distortion at low frequency, and the dry electrodes based on cowhide had better performance. In the skin-electrode impedance test, the air gap impedance of our flexible electrodes was larger than the gel impedance of the standard Ag/AgCl electrode. However, the impedance of the prepared electrodes (the electrode based on the top grain layer: from 180.85 kΩ to 53.67 kΩ (Figure 6a, f = 200 Hz), the electrode based on the split layer: from 732.06 kΩ to 98.37 kΩ (Figure 6a, f = 200 Hz) was significantly reduced to the same level as the standard Ag/AgCl electrode (52.51 kΩ (Figure 6a, f = 200 Hz)) after applying a non-allergenic lotion. In the ECG test, the SNR of the ECG signals measured by the three electrodes in sitting posture shows no significant difference. The signal-to-noise ratio (SNR) of the electrodes based on the top grain layer and the split layer and the standard electrode was 9.446 dB, 8.059 dB, and 6.747 dB in the standing posture, respectively. In the power spectrum of ECG, the ECG measured by all the three electrodes can reflect the signal characteristics (such as heart rate). So, cowhide is a potential electrode substrate material.

## Figures and Tables

**Table 1:** The results of the resistance measurement.

Type of Electrodes	Red Points	Blue Points	White Points	Green Points	The AVG and SD of the Resistance of Four Group Points
① ^1^	0.23 Ω	0.20 Ω	0.27 Ω	0.30 Ω	(0.25 ± 0.04) Ω
② ^2^	0.27 Ω	0.33 Ω	0.30 Ω	0.23 Ω	(0.28 ± 0.04) Ω

^1^ ① represents the electrode based on the top grain layer of cowhide.; ^2^ ② represents the electrode based on the split layer of cowhide.

**Table 2:** Equivalent circuit parameters of skin-electrode interface.

Type of Electrodes	Goodness of Fit	R_d_ (kΩ)	R_e_ (kΩ)	C_e_ (nF)	R_I_ (kΩ)	C_I_ (nF)	R_i_ (kΩ)	C_i_ (nF)	Z_1_ (kΩ)	Z_2_ (kΩ)	Z_3_ (kΩ)	Z_4_ (kΩ)	Z (kΩ)
① ^1^	0.99	1.92	252.00	41.90	1.92	N.A.^6^	16.80	117.00	1.92	152.00	1.92	16.30	172.00
② ^2^	0.98	2.72	124.00	25.80	79.30	6.22	1600.00	13,60	2.72	115.00	79.20	542.00	739.00
③ ^3^	0.92	1.89	122.00	2.01	507.00	5.56	5,000,000.00	5.66	1.89	122.00	478.00	1410.00	2010.00
④ ^4^	0.99	1.86	157.00	57.50	1.86	N.A.^6^	24.20	89.30	1.86	104.00	1.86	23.30	131.00
⑤ ^5^	0.98	2.45	411.00	47.10	2.63	N.A.^6^	316.00	13.10	2.45	156.00	2.63	280.00	442.00

^1^ ① represents the standard Ag/AgCl electrode; ^2^ ② represents the electrode based on the top grain layer of cowhide; ^3^ ③ represents the electrode based on the split layer of cowhide; ^4^ ④ represents the electrode based on the top grain layer with lotion; ^5^ ⑤ represents the electrode based on the split layer with lotion; ^6^ There is no C_I_ in the equivalent circuit of wet electrode, so C_I_ is not applicable.

## Data Availability

The data presented in this study are available on request from the corresponding author. The data are not publicly available due to privacy and ethical.

## Supplementary Materials

The following are available online at https://www.mdpi.com/article/10.3390/bios11040101/s1, Figure S1: the skin electrode impedance result of the second subject, Figure S2: the skin electrode impedance result of the third subject, Figure S3: the skin electrode impedance result of the fourth subject, Figure S4: the skin electrode impedance result of the fifth subject, Figure S5: the skin electrode impedance result of the sixth subject, Figure S6: the ECG result of the second subject, Figure S7: the ECG result of the third subject, Figure S8: the ECG result of the fourth subject, Figure S9: the ECG result of the fifth subject, Figure S10: the ECG result of the sixth subject.
