# Supplementary material for: A Novel Wearable Flexible Dry Electrode Based on Cowhide for ECG Measurement
Source: Biosensors (Basel). 2021 Apr 1;11(4):101. doi: 10.3390/bios11040101 (PMC8065990; doi:10.3390/bios11040101)
Supplement: Supplementary file 1 [file biosensors-11-00101-s001.pdf]

biosensors

SUPPORTING INFORMATION

A Novel Wearable Flexible Dry Electrode Based on Cowhide for ECG Measurement of a young woman

Yiping Huang [1], Yatong Song [2], Li Gou [3] and Yuanwen Zou [4,*]

[1] College of Biomedical Engineering, Sichuan University, Chengdu 610065, China; hyp@stu.scu.edu.cn
[2] College of Biomedical Engineering, Sichuan University, Chengdu 610065, China; songyatong@stu.scu.edu.cn
[3] College of Biomedical Engineering, Sichuan University, Chengdu 610065, China; gouli@scu.edu.cn
[4] College of Biomedical Engineering, Sichuan University, Chengdu 610065, China; zyw@scu.edu.cn
* Correspondence: zyw@scu.edu.cn; Tel.: 86-28-85405140

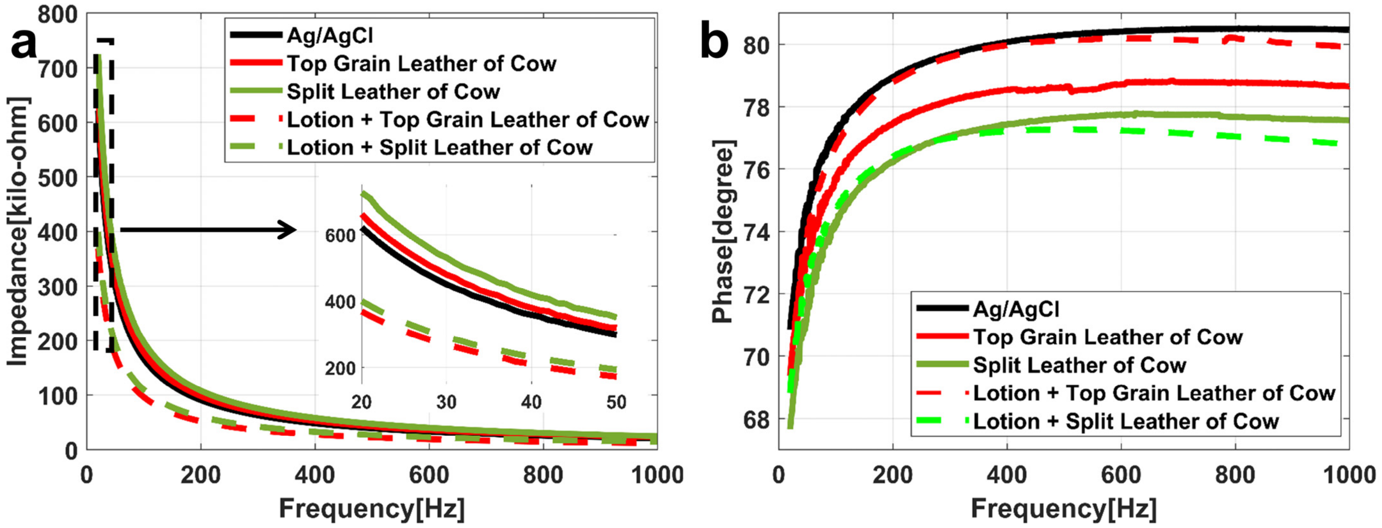

Figure S1. Diagram of (a) skin-electrode impedance-frequency and (b) skin-electrode phase-frequency of the second subject. The frequency range is 20-1000 Hz, each electrode is indicated by one color, and the application of lotion is indicated by a dotted line.

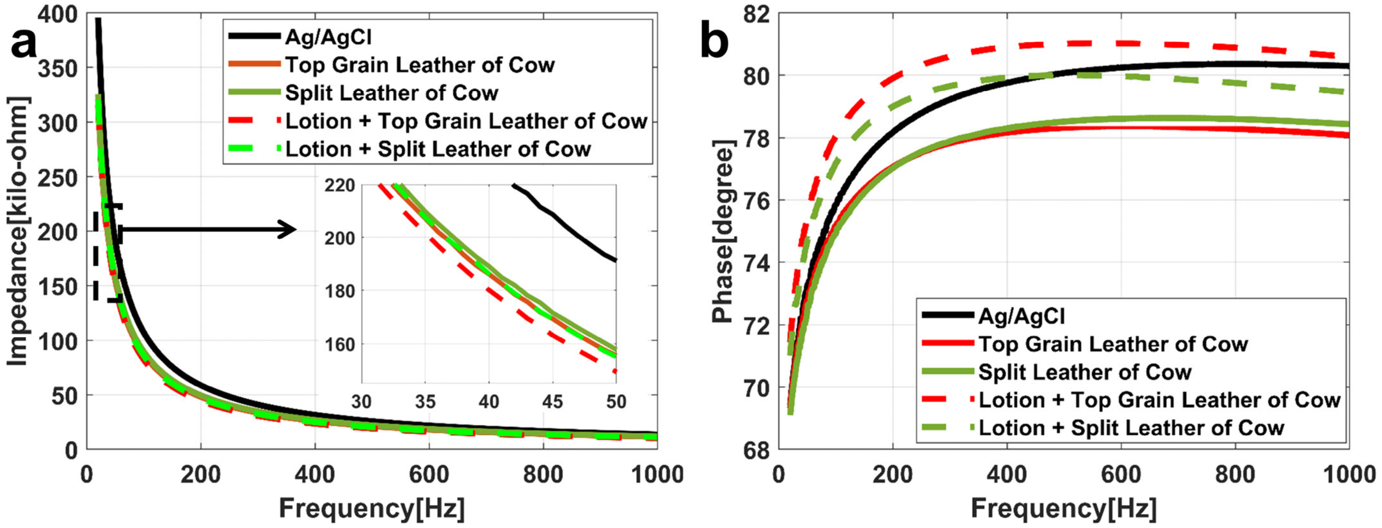

Figure S2. Diagram of (a) skin-electrode impedance-frequency and (b) skin-electrode phase-frequency of the third subject. The frequency range is 20-1000 Hz, each electrode is indicated by one color, and the application of lotion is indicated by a dotted line. (The impedance of the electrode based on cowhide of the subject is lower than that of the standard electrode, which may be due to the skin stratum corneum is removed frequently and body lotion was used often in the daily life of the third subject. These behaviors can cause thin stratum corneum and higher moisture content of skin, resulting in the final skin electrode impedance test results.)

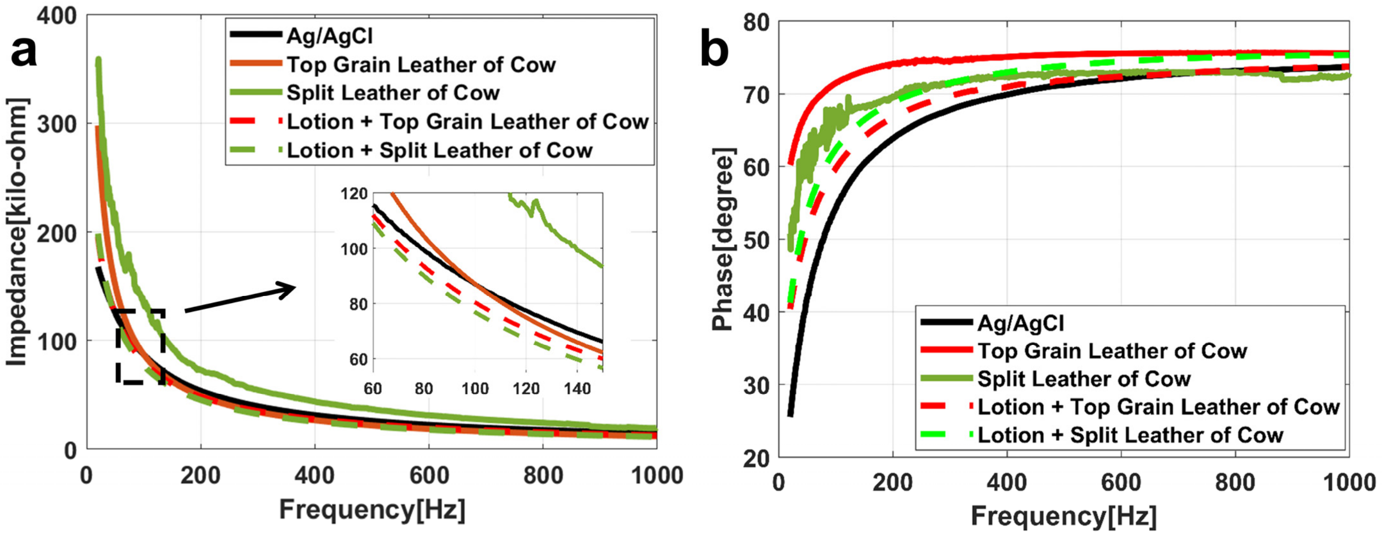

Figure S3. Diagram of (a) skin-electrode impedance-frequency and (b) skin-electrode phase-frequency of the fourth subject. The frequency range is 20-1000 Hz, each electrode is indicated by one color, and the application of lotion is indicated by a dotted line. (Compared with other subjects, the overall impedance of the subject was lower, and the impedance of the electrode based on the top grain layer of cowhide was lower than that of the standard electrode when the frequency was more than 100Hz, which may be related to that the skin stratum corneum was removed deeply by scrubing and washing skin forcefully few days ago.)

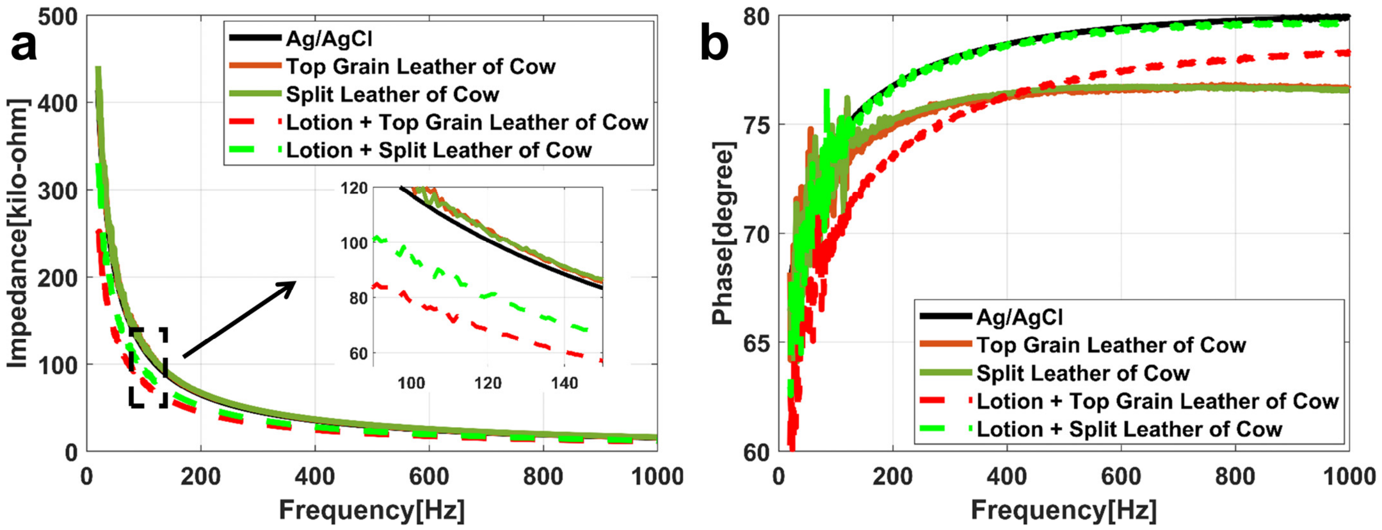

Figure S4. Diagram of (a) skin-electrode impedance-frequency and (b) skin-electrode phase-frequency of the fifith subject. The frequency range is 20-1000 Hz, each electrode is indicated by one color, and the application of lotion is indicated by a dotted line.

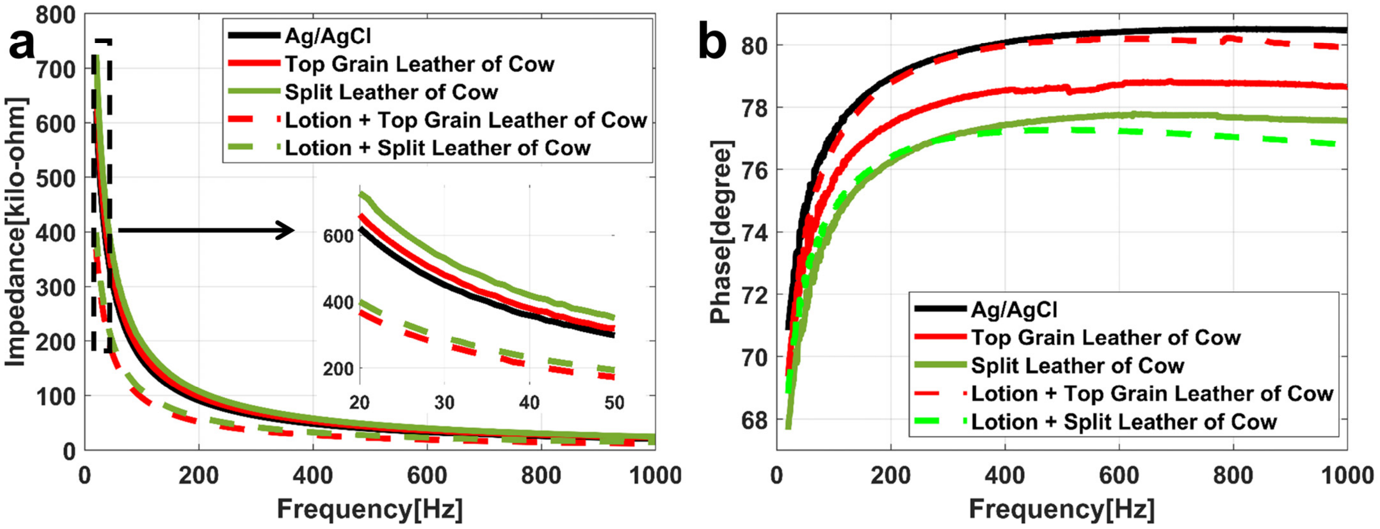

Figure S5. Diagram of (a) skin-electrode impedance-frequency and (b) skin-electrode phase-frequency of the sixth subject. The frequency range is 20-1000 Hz, each electrode is indicated by one color, and the application of lotion is indicated by a dotted line.

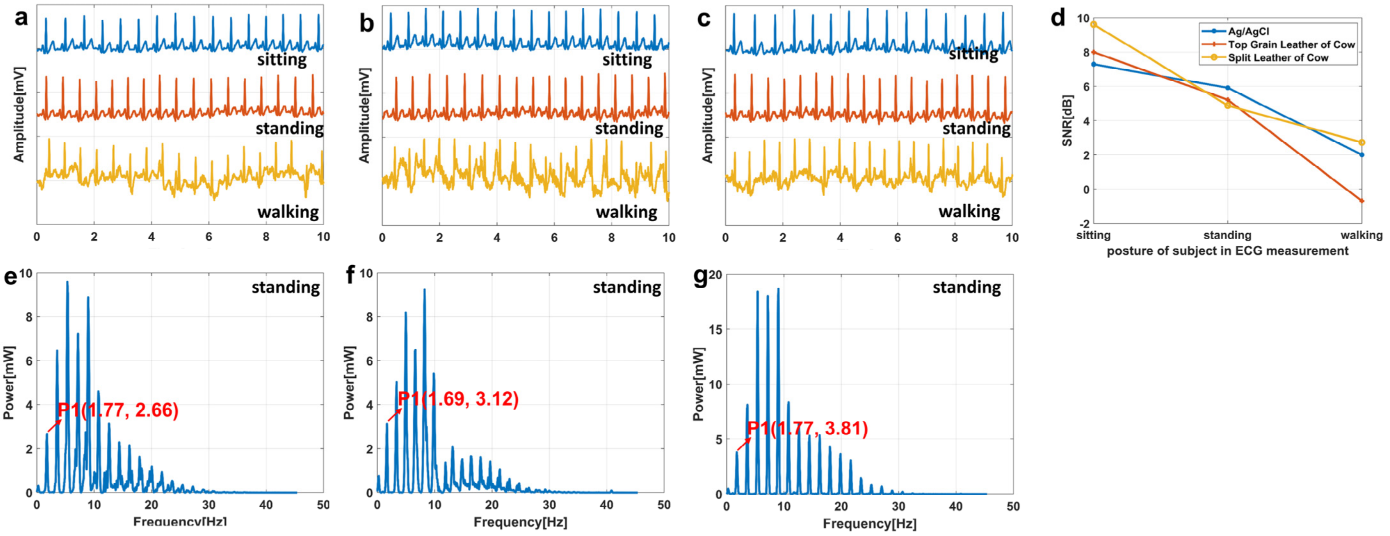

Figure S6. The ECG result of the second subject. The ECG of (a) the standard Ag/AgCl electrode and (b) (c) the electrodes based on the top grain layer and split layer. (d) Signal-to-noise ratio (SNR) diagram. The power spectrum of the standing posture of (e) the standard Ag/AgCl electrode (f) the electrode based on the top grain layer and (g) the electrode based on the split layer. (The heart rates of the subject are 106.2, 101.4 and 106.2 beats per minute, which is normal when the subject was obviously nervous.)

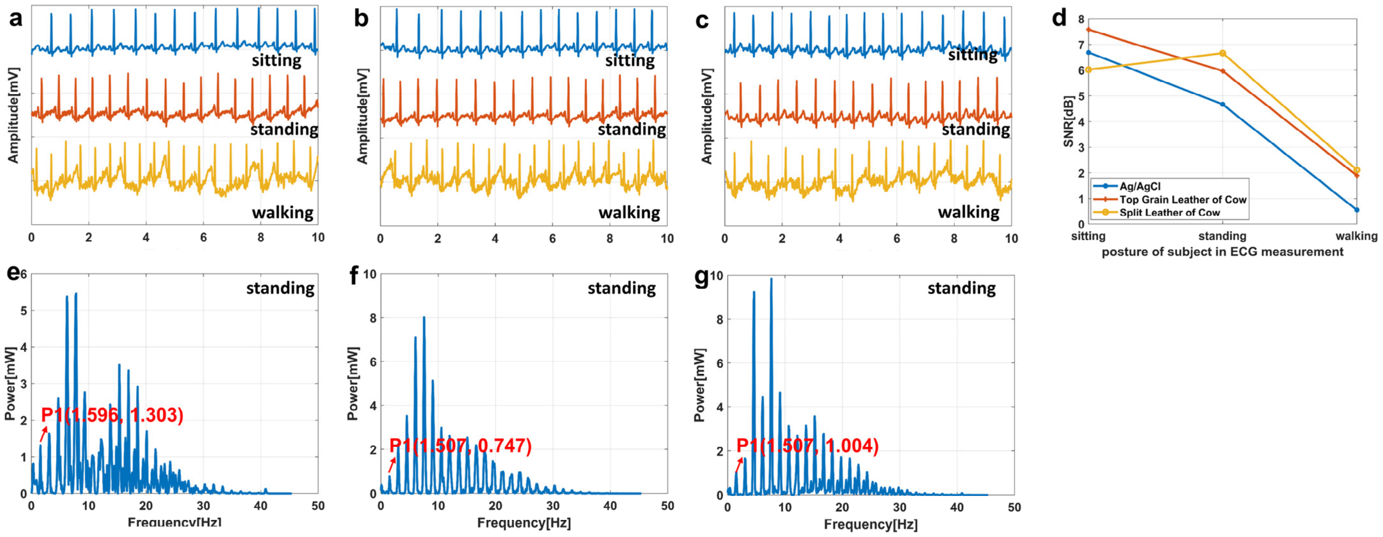

Figure S7. The ECG result of the third subject. The ECG of (a) the standard Ag/AgCl electrode and (b) (c) the electrodes based on the top grain layer and split layer. (d) Signal-to-noise ratio (SNR) diagram. The power spectrum of the standing posture of (e) the standard Ag/AgCl electrode (f) the electrode based on the top grain layer and (g) the electrode based on the split layer. (The heart rates of the subject are 95.76, 90.42 and 95.76 beats per minute, which is normal.)

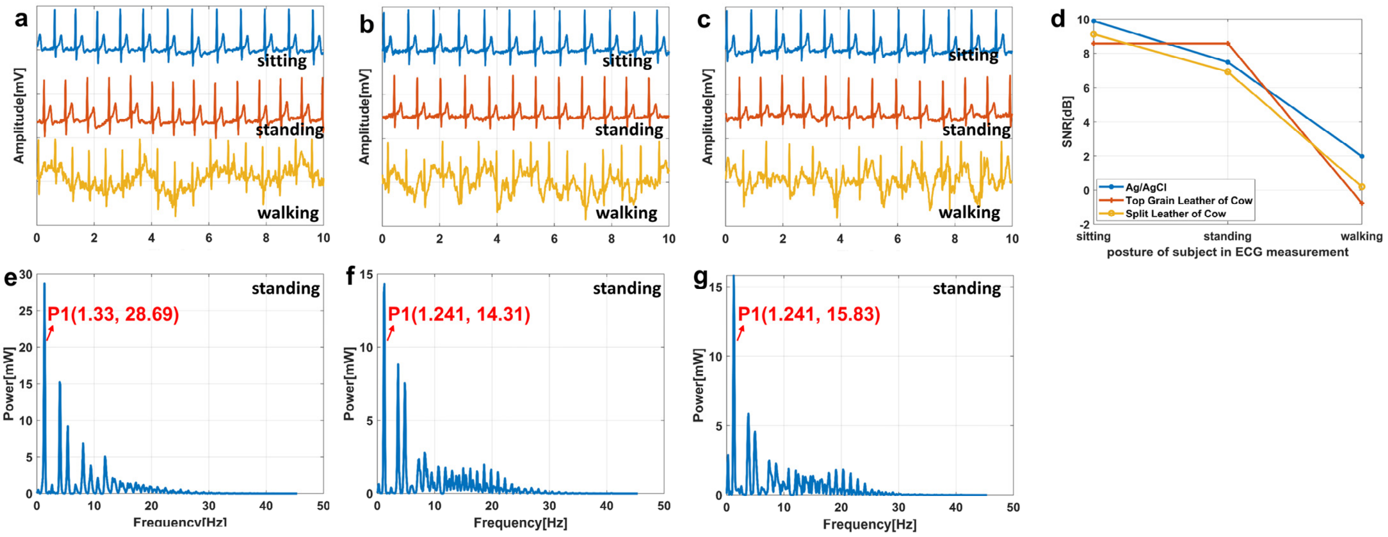

Figure S8. The ECG result of the fourth subject. The ECG of (a) the standard Ag/AgCl electrode and (b) (c) the electrodes based on the top grain layer and split layer. (d) Signal-to-noise ratio (SNR) diagram. The power spectrum of the standing posture of (e) the standard Ag/AgCl electrode (f) the electrode based on the top grain layer and (g) the electrode based on the split layer. (The heart rates of the subject are 79.8, 74.46 and 74.46 beats per minute, which is normal.)

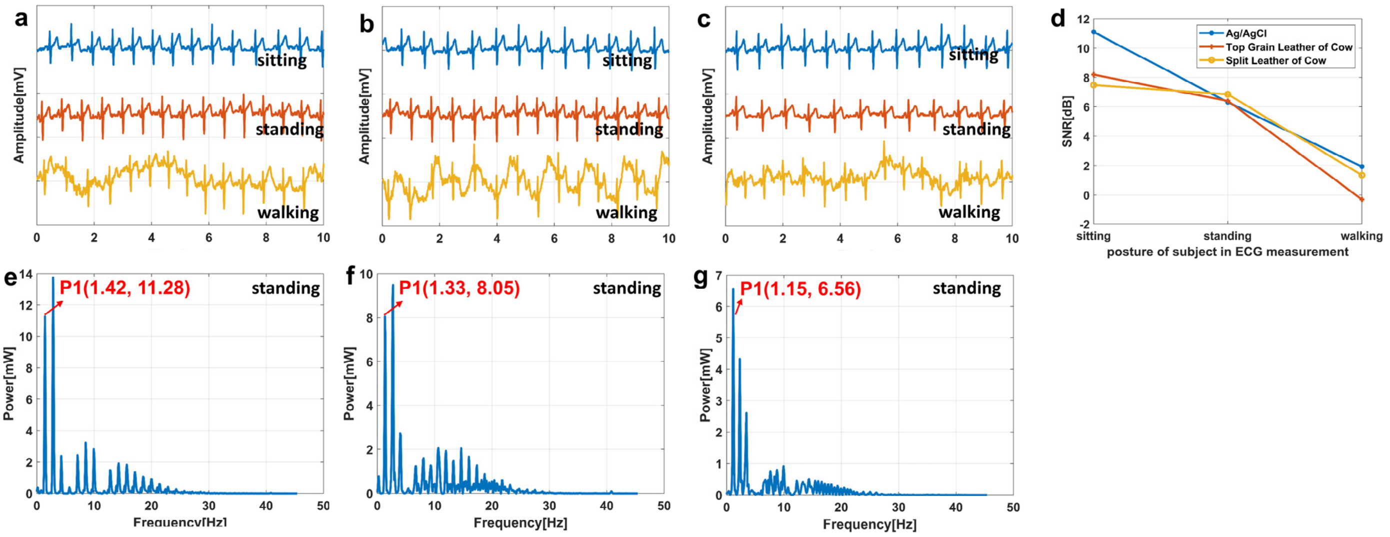

Figure S9. The ECG result of the fifth subject. The ECG of (a) the standard Ag/AgCl electrode and (b) (c) the electrodes based on the top grain layer and split layer. (d) Signal-to-noise ratio (SNR) diagram. The power spectrum of the standing posture of (e) the standard Ag/AgCl electrode (f) the electrode based on the top grain layer and (g) the electrode based on the split layer. (The heart rates of the subject are 85.2, 79.8 and 69.0 beats per minute, which is normal.)

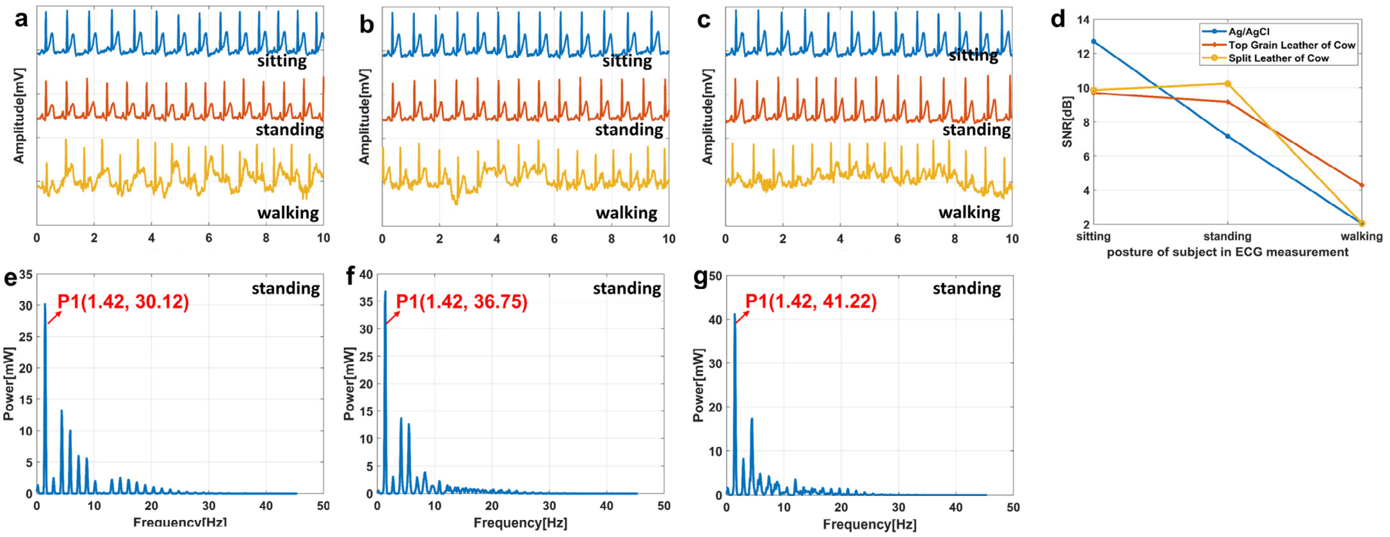

Figure S10. The ECG result of the sixth subject. The ECG of (a) the standard Ag/AgCl electrode and (b) (c) the electrodes based on the top grain layer and split layer. (d) Signal-to-noise ratio (SNR) diagram. The power spectrum of the standing posture of (e) the standard Ag/AgCl electrode (f) the electrode based on the top grain layer and (g) the electrode based on the split layer. (The heart rates of the subject are all 85.2 beats per minute, which is normal.)
